# Supplementary material for: Genomic loci involved in sensing environmental cues and metabolism affect seasonal coat shedding in Bos taurus and Bos indicus cattle
Source: G3 (Bethesda). 2023 Dec 13;14(2):jkad279. doi: 10.1093/g3journal/jkad279 (PMC10849337; doi:10.1093/g3journal/jkad279)
Supplement: jkad279_Supplementary_Data [file jkad279_supplementary_data.zip › File_S1_G3-2023-404501.pdf]

File S1

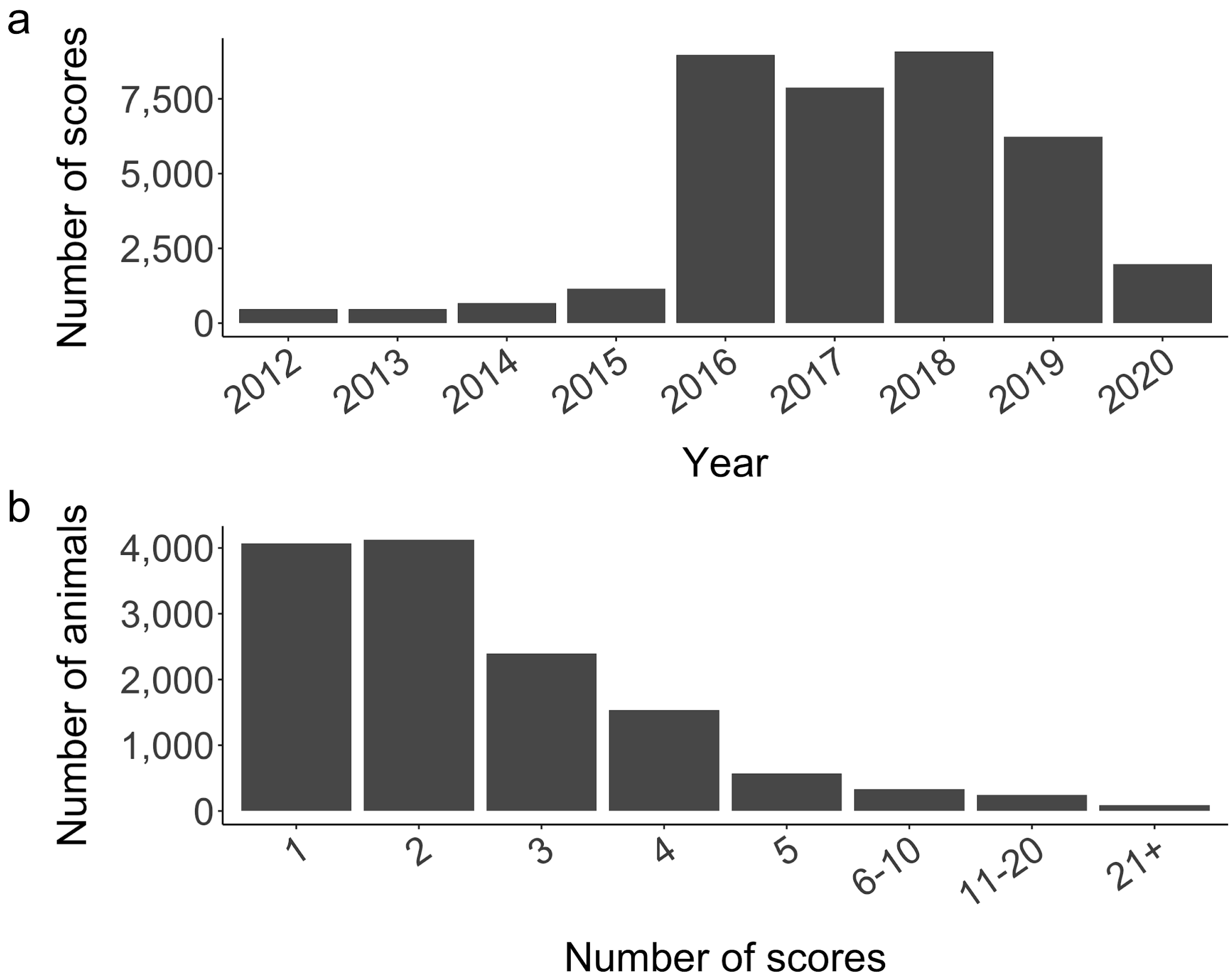

Figure S1. Counts of **(A)** hair shedding scores per year and **(B)** scores per animal across all years.

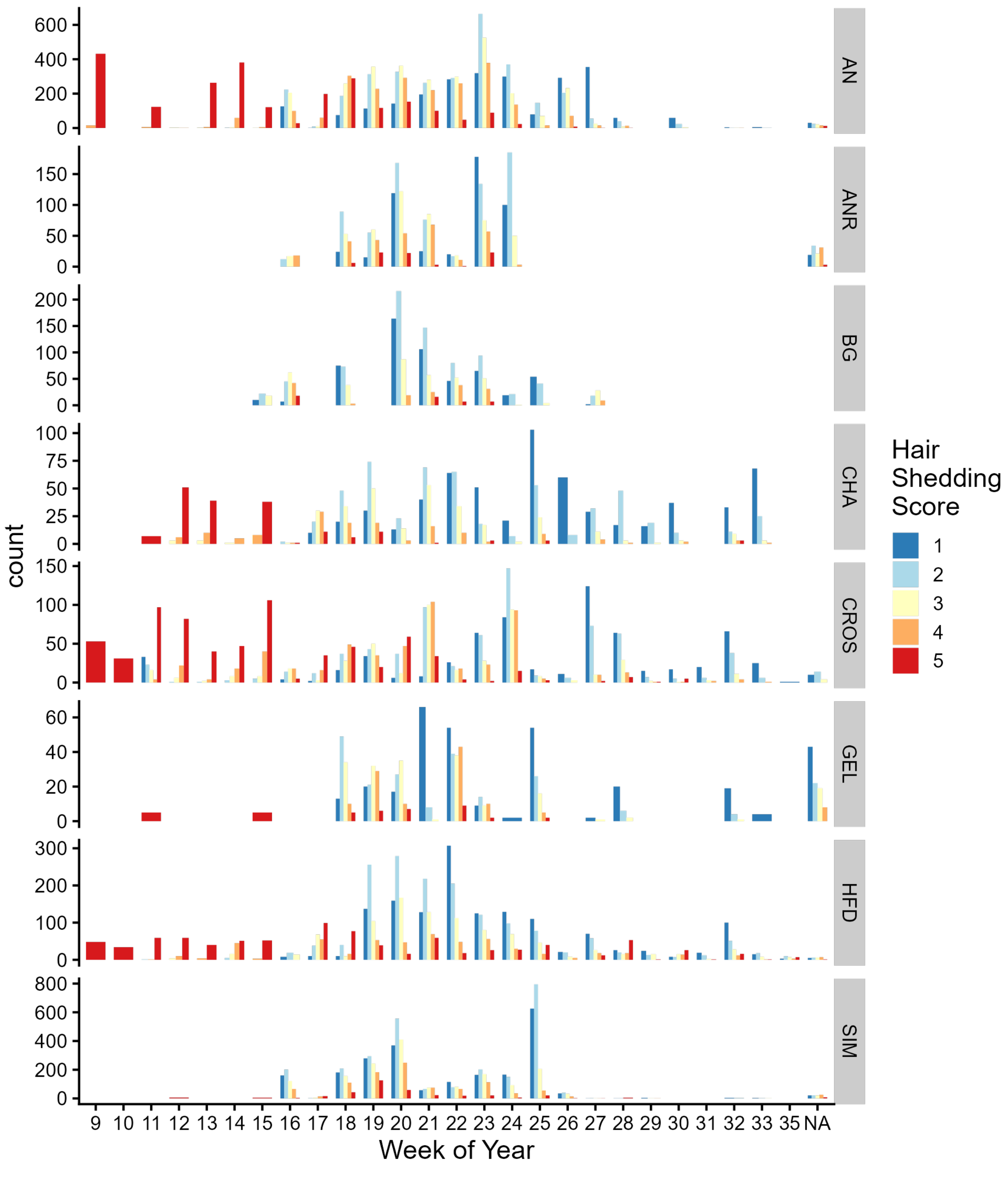

Figure S2. Bar graph of counts of hair shedding scores per breed across all years. Most of the data is from from week 17 (beginning of May) to week 24 (the end of June). Braunvieh, Chianina, Simbrah, Maine-Anjou, and Shorthorn were excluded from the plot due to small sample sizes.
Breed codes as follows: AN – Angus, ANR – Red Angus, BG – Brangus, CHA – Charolais, CROS – Crossbred, GEL – Gelbvieh, HFD – Hereford, SIM – Simmental.

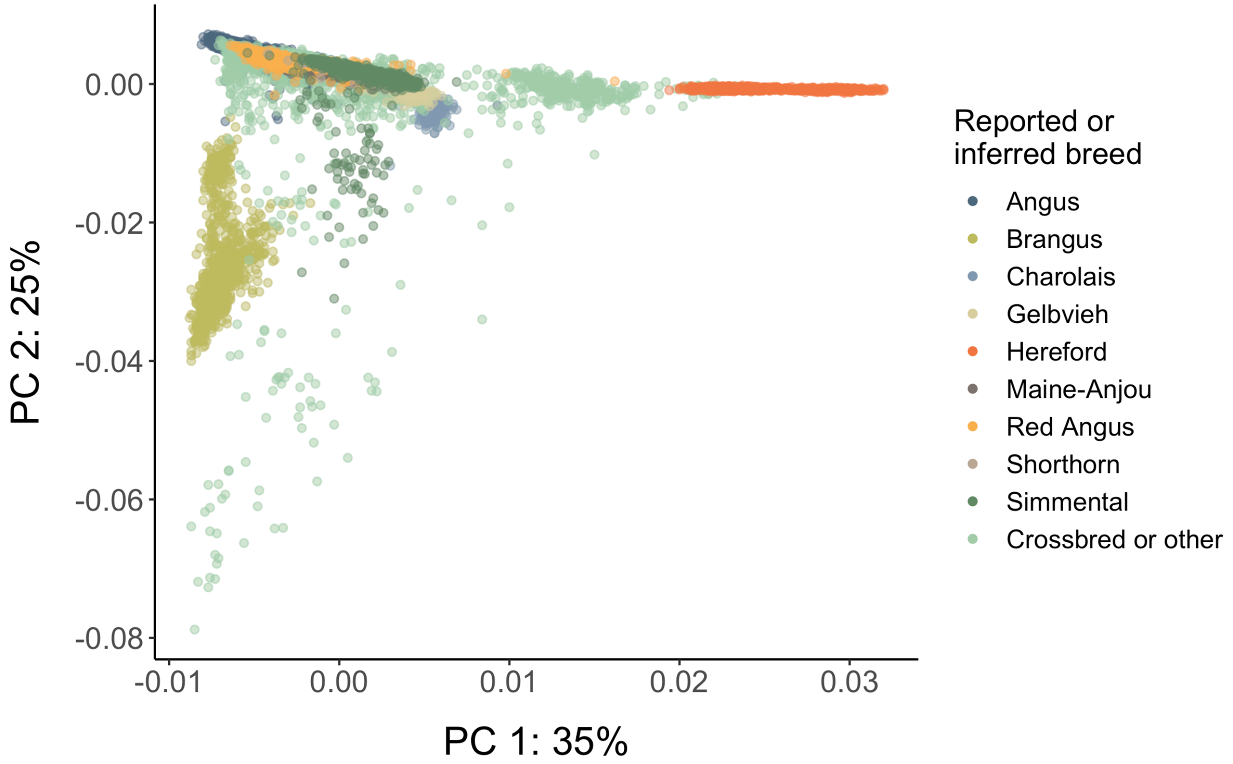

Figure S3. Principal components 1 and 2. For the purposes of this visualization, Angus, Hereford, Red Angus, Simmental, and Gelbvieh animals with at least ⅝ pedigree ancestry assigned to the given breed based on pedigree estimates were included in that breed. Animals with unknown ancestry, less than ⅝ pedigree ancestry assigned to one breed, or of a breed not listed above were called “Crossbred or other”.

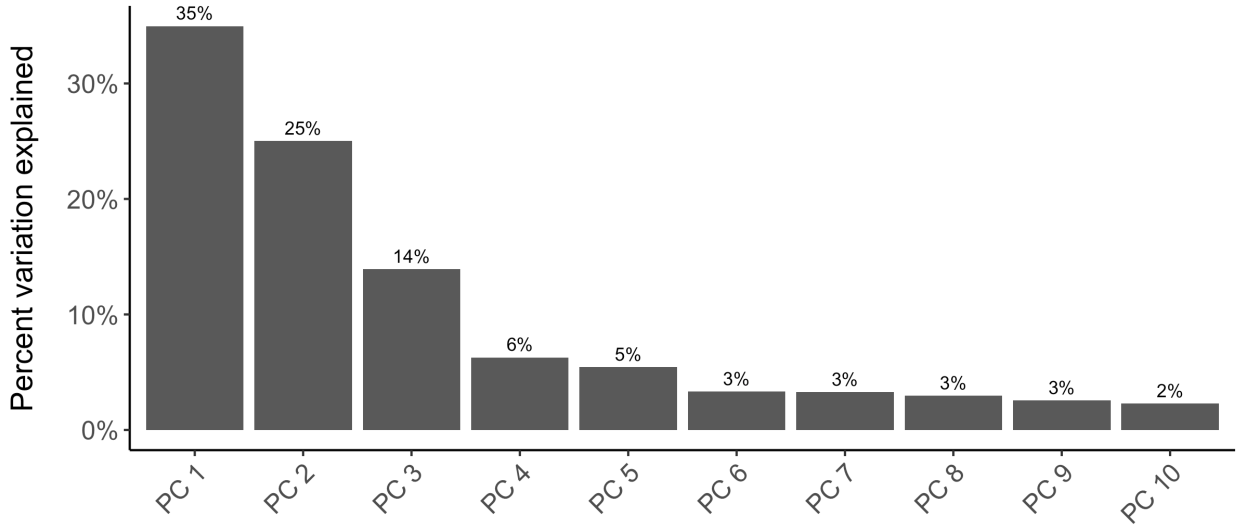

Figure S4. Principal components 1 and 2 explain 65% of variation in a principal component analysis of all 11,560 genotyped animals in the dataset.

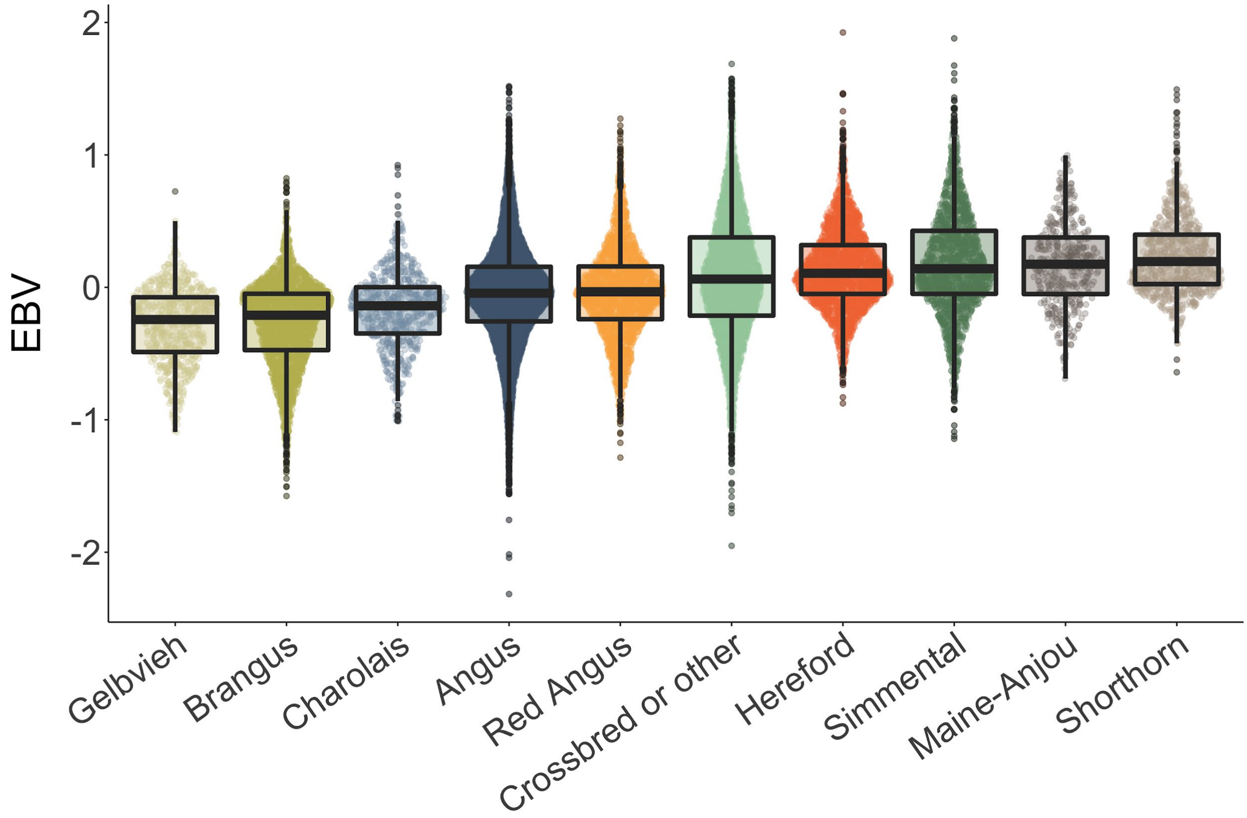

Figure S5. Comparison of EBVs from the full dataset by breed. For the purposes of this summarization, Angus, Hereford, Red Angus, Simmental, and Gelbvieh animals with at least ⅝ pedigree ancestry assigned to the given breed based on pedigree estimates were included in that breed. Animals with unknown ancestry, less than ⅝ pedigree ancestry assigned to one breed, or of a breed not listed above were called “Crossbred or other.”

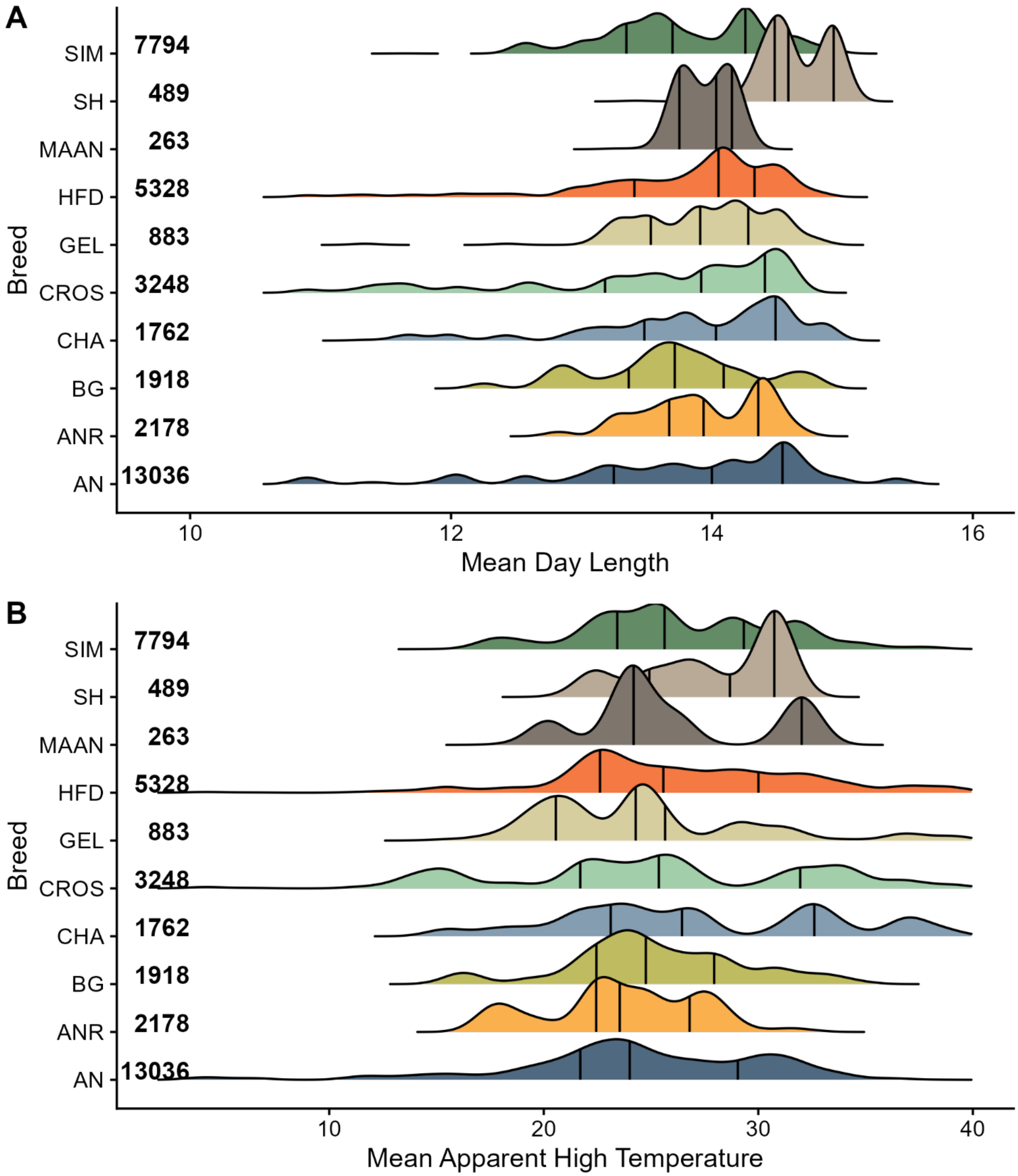

Figure S6. Density ridge plot of **(A)** Mean day length (the average number of sunlight hours for the 30 days prior to the hair shedding observation) and **(B)** Mean apparent high temperature (combination of humidity and air temperature averaged across the 30 days prior to the hair shedding observation). Data is shown across years.

Table S1. Evaluation of breeding values in the full and breed-specific datasets across 10 iterations.

Prediction accuracy				
**Dataset**	**Minimum**	**Mean**	**Maximum**	**SD**
Angus	0.585	0.594	0.601	0.006
Brangus	0.514	0.524	0.536	0.007
Hereford	0.498	0.520	0.545	0.013
IGS	0.653	0.663	0.676	0.007
Full dataset	0.657	0.665	0.674	0.006
Dispersion ($b^{v}_{w,p}$)				
**Dataset**	**Minimum**	**Mean**	**Maximum**	**SD**
Angus	0.931	1.007	1.089	0.055
Brangus	0.903	1.014	1.116	0.072
Hereford	0.853	1.027	1.252	0.133
IGS	0.964	1.036	1.154	0.049
Full dataset	0.975	1.009	1.041	0.021
Bias (Δ_p)				
**Dataset**	**Minimum**	**Mean**	**Maximum**	**SD**
Angus	0.000	0.091	1.356	0.107
Brangus	0.000	0.069	0.964	0.091
Hereford	0.000	0.072	1.779	0.088
IGS	0.000	0.094	1.397	0.113
Full dataset	0.000	0.084	1.966	0.097

Table S2. BLUEs for toxic fescue grazing status across four increasingly complex models quantifying the effects of daily sunlight duration and temperature on hair shedding with approximated standard errors in parentheses. “Fall calving” BLUEs are relative to “spring calving” BLUE = 0, “not grazing toxic fescue” BLUEs are relative to “grazing toxic fescue” BLUE = 0, and age group BLUEs are relative to “age: yearling” BLUE = 0.

Model	Fall calving	Not grazing toxic fescue	Age: 2-3 year olds	Age: 4-9 year olds	Age: 10+
Day length + covariates	-0.122 (0.020)	-0.222 (0.022)	0.091 (0.019)	-0.057 (0.021)	-0.206 (0.028)
Temperature + covariates	0.001 (0.019)	-0.055 (0.022)	0.106 (0.019)	-0.077 (0.021)	-0.231 (0.027)
Day length + temperature + covariates	-0.056 (0.019)	-0.124 (0.022)	0.100 (0.018)	-0.067 (0.021)	-0.215 (0.027)
Day length + temperature + day length*temperature + covariates	-0.061 (0.019)	-0.136 (0.022)	0.102 (0.018)	-0.061 (0.021)	-0.208 (0.027)

Table S3. Gene set enrichment results. Considered data sources included GO biological processes, GO cellular components, GO molecular functions, KEGG pathways, Reactome pathways, CORUM pro, and disease phenotypes annotated by the Human Phenotype Ontology database. "Intersecting genes" are those genes in the set that were annotated to the term.

Dataset	Term	Source	Adjusted p-value	Intersecting genes
Main effect - DEBVs	regulation of cell death	GO:BP	2.19E-03	*ALX3; ZC3H12A; KITLG; CRADD; ITGA5; ZNF385A; ALB; BTC; PACRG; EEF1A2; CTSZ; PTPMT1; NDUFS3; MTCH2; OSGIN1; GH1; SOX9; MITF; SOX4; E2F3*
Main effect - DEBVs	regulation of apoptotic process	GO:BP	8.72E-03	*ALX3; ZC3H12A; KITLG; CRADD; ITGA5; ZNF385A; ALB; BTC; EEF1A2; PTPMT1; NDUFS3; MTCH2; OSGIN1; GH1; SOX9; MITF; SOX4; E2F3*
Main effect - DEBVs	regulation of programmed cell death	GO:BP	1.28E-02	*ALX3; ZC3H12A; KITLG; CRADD; ITGA5; ZNF385A; ALB; BTC; EEF1A2; PTPMT1; NDUFS3; MTCH2; OSGIN1; GH1; SOX9; MITF; SOX4; E2F3*
Main effect - DEBVs	PTK6 Down-Regulation	REAC	1.76E-02	*SRMS; PTK6*
Main effect - DEBVs	cell death	GO:BP	1.77E-02	*ALX3; AHCYL1; ZC3H12A; KITLG; CRADD; ITGA5; ZNF385A; ALB; BTC; PACRG; EEF1A2; CTSZ; PTPMT1; NDUFS3; MTCH2; OSGIN1; GH1; SOX9; MITF; SOX4; E2F3*
Main effect - meta-analysis	White forelock	HP	8.24E-04	*KITLG; EDN3; MITF; TFAP2A*
Main effect - meta-analysis	Patchy hypopigmentation	HP	4.96E-03	*KITLG; EDN3; MITF; TFAP2A*

	of hair			
Main effect - meta-analysis	regulation of cell death	GO:BP	8.98E-03	*ALX3; UTP11; ZC3H12A; KITLG; DUSP6; CRADD; ITGA5; ZNF385A; ALB; BTC; PACRG; CTSZ; MTCH2; OSGIN1; SOX9; MITF; SOX4; E2F3; TFAP2A*
Main effect - meta-analysis	cell death	GO:BP	1.77E-02	*ALX3; AHCYL1; UTP11; ZC3H12A; KITLG; DUSP6; CRADD; ITGA5; ZNF385A; ALB; BTC; PACRG; CTSZ; MTCH2; OSGIN1; ERN1; SOX9; MITF; SOX4; E2F3; TFAP2A*
Main effect - meta-analysis	receptor ligand activity	GO:MF	3.21E-02	*KITLG; BTC; EDN3; ENSBTAG00000009943; XCL1; OSGIN1; TAFA4; ENSBTAG00000049502; TAFA1; ENSBTAG00000048492*
Main effect - meta-analysis	regulation of apoptotic process	GO:BP	3.49E-02	*ALX3; UTP11; ZC3H12A; KITLG; DUSP6; CRADD; ITGA5; ZNF385A; ALB; BTC; MTCH2; OSGIN1; SOX9; MITF; SOX4; E2F3; TFAP2A*
Main effect - meta-analysis	signaling receptor activator activity	GO:MF	3.67E-02	*KITLG; BTC; EDN3; ENSBTAG00000009943; XCL1; OSGIN1; TAFA4; ENSBTAG00000049502; TAFA1; ENSBTAG00000048492*
Main effect - meta-analysis	regulation of programmed cell death	GO:BP	4.97E-02	*ALX3; UTP11; ZC3H12A; KITLG; DUSP6; CRADD; ITGA5; ZNF385A; ALB; BTC; MTCH2; OSGIN1; SOX9; MITF; SOX4; E2F3; TFAP2A*
COJO	extracellular space	GO:CC	1.70E-02	*ALB; AFP; AFM; EMILIN1; CTSZ; ENSBTAG00000009943; XCL1; WFDC1; TAFA4; ENSBTAG00000049502; CRISP1; TNXB; ENSBTAG00000006864*
COJO	Notch binding	GO:MF	2.10E-02	*GALNT11; CNTN6; NOTCH4*
COJO	myeloid leukocyte migration	GO:BP	2.73E-02	*EMILIN1; ENSBTAG00000009943; XCL1; TAFA4; ENSBTAG00000049502; AGER*

GxE - day length	keratin filament	GO:CC	2.49E-04	*KRTAP10-8; ENSBTAG00000053056; KRTAP12-2; ENSBTAG00000051778; ENSBTAG00000050038; KRT74; KRT5; KRT6A; KRT6B; ENSBTAG00000040019; FAM83H*
GxE - day length	Onychogryposis of toenails	HP	6.23E-03	*KRT6A; KRT6B; ENSBTAG00000040019; PLEC*
GxE - day length	intermediate filament	GO:CC	1.57E-02	*KRTAP10-8; ENSBTAG00000053056; KRTAP12-2; ENSBTAG00000051778; ENSBTAG00000050038; KRT74; KRT5; KRT6A; KRT6B; ENSBTAG00000040019; PLEC; FAM83H*
GxE - day length	PFKL-PFKM-PFKP complex	CORUM	1.88E-02	*PFKL; PFKP*
GxE - day length	intermediate filament cytoskeleton	GO:CC	2.50E-02	*KRTAP10-8; ENSBTAG00000053056; KRTAP12-2; ENSBTAG00000051778; ENSBTAG00000050038; KRT74; KRT5; KRT6A; KRT6B; ENSBTAG00000040019; EXOSC4; PLEC; FAM83H*
GxE - day length	Palmoplantar blistering	HP	2.80E-02	*KRT5; KRT6A; KRT6B; ENSBTAG00000040019*
GxE - day length	Paronychia	HP	3.54E-02	*KRT6A; KRT6B; ENSBTAG00000040019; LAMC2; RETREG1*
GxE - day length	Metabolism	REAC	3.80E-02	*LIPH; PFKL; HLCS; CPS1; ALDH9A1; MGST3; ACP6; PRKAB2; HMGCS2; GNG5; PSMB2; NAMPT; AKR1D1; LUM; DCN; ARSJ; SCD5; ACER2; GLDC; PLPP6; CGA; PLCB2; GPAT2; PSMB7; NUP188; MIGA2; GPC5; PFKP; PARP10; PYCR3; SLC25A32; PIK3C2B; ACBD6; NMNAT2; FLVCR1; NCOR2; SLC7A5; CA5A; CMBL; CYP1A2; CYP1A1; TKT; MOCOS; ACSM5;*

				CRYM; MLXIPL; HGSNAT; NDST2
GxE - day length	keratinization	GO:BP	4.83E-02	*LCE1B; ENSBTAG00000052594; ENSBTAG00000053743; ENSBTAG00000054620; ENSBTAG00000050431*

Table S4. QTL enrichment results. "N annotations" represent the number of Animal QTLdb annotations.

Dataset	QTL name	Adjusted p-value	N annotations	N SNPs	SNPs
Main effect - DEBVs	White spotting	8.35E-30	19	6	22:31,647,147; 22:32,120,539; 22:32,204,981; 22:32,208,077; 22:32,209,610; 22:32,979,644
Main effect - DEBVs	Body weight (mature)	7.28E-05	6	24	19:59,031,592; 19:59,042,109; 19:59,049,072; 19:59,058,798; 19:59,060,008; 19:59,062,908; 19:59,066,604; 19:59,069,136; 19:59,083,455; 19:59,084,200; 19:59,087,943; 19:59,091,866; 19:59,120,248; 19:59,133,155; 19:59,173,133; 19:59,179,679; 19:59,307,342; 19:59,309,672; 19:59,316,368; 19:59,327,548; 19:59,371,948; 19:59,379,387; 19:59,382,908; 19:59,393,728
Main effect - DEBVs	Clinical mastitis	2.11E-04	8	6	6:88,497,883; 13:57,010,672; 13:57,021,032; 13:57,029,389; 13:57,037,937; 13:57,744,564
Main effect - DEBVs	Body weight (18 months)	1.07E-02	2	18	5:18,834,893; 5:18,837,012; 5:18,837,973; 5:18,840,051; 5:18,845,572; 5:18,853,702; 5:18,861,017; 5:18,861,915; 5:18,863,193; 5:18,864,340; 5:18,865,142; 5:18,866,748; 5:18,876,734; 5:18,889,693; 5:18,924,994; 22:32,663,366; 22:32,718,597; 22:32,721,303
Main effect - DEBVs	Sire conception rate	2.03E-02	2	7	9:42,516,598; 13:57,939,566; 13:57,940,287; 13:57,958,615; 13:57,962,862; 13:57,971,981; 13:57,984,480
Main effect - DEBVs	Eye area pigmentation	3.82E-02	2	3	22:32,120,539; 22:32,663,366; 22:32,718,597
Main effect - meta-analysis	White spotting	6.34E-41	25	12	22:31,346,055; 22:31,601,001; 22:31,608,442; 22:31,647,147; 22:31,651,707; 22:31,653,105; 22:31,807,382; 22:32,120,539; 22:32,204,981; 22:32,208,077; 22:32,209,610; 22:32,979,644

Main effect - meta-analysis	Body weight (mature)	1.10E-05	7	28	5:22,351,623; 19:59,005,190; 19:59,009,634; 19:59,010,199; 19:59,011,273; 19:59,031,592; 19:59,042,109; 19:59,058,798; 19:59,062,908; 19:59,066,604; 19:59,069,136; 19:59,091,866; 19:59,103,428; 19:59,112,204; 19:59,120,248; 19:59,133,155; 19:59,167,705; 19:59,168,801; 19:59,173,133; 19:59,175,249; 19:59,179,679; 19:59,202,424; 19:59,307,342; 19:59,309,672; 19:59,316,368; 19:59,382,908; 19:59,391,271; 19:59,393,728
Main effect - meta-analysis	Clinical mastitis	5.37E-03	7	4	6:88,493,833; 6:88,497,883; 13:57,010,672; 13:57,037,937
Main effect - meta-analysis	Body weight (18 months)	2.45E-02	2	18	5:18,834,893; 5:18,837,012; 5:18,837,973; 5:18,840,051; 5:18,845,572; 5:18,853,702; 5:18,861,017; 5:18,861,915; 5:18,863,193; 5:18,864,340; 5:18,865,142; 5:18,866,748; 5:18,876,734; 5:18,889,693; 5:18,924,994; 22:32,663,366; 22:32,718,597; 22:32,721,303
Main effect - meta-analysis	Hoof and leg disorders	4.29E-02	2	4	22:31,608,442; 22:31,647,147; 22:31,651,707; 22:31,653,105
COJO	Interval from first to last insemination	2.83E-32	26	5	17:68,938,350; 17:69,096,404; 17:69,149,498; 17:69,155,480; 17:69,157,843
COJO	Non-return rate	3.43E-14	26	5	17:68,938,350; 17:69,096,404; 17:69,149,498; 17:69,155,480; 17:69,157,843
COJO	Calf size	3.07E-03	3	4	13:57,182,321; 13:57,201,678; 17:69,155,480; 17:69,157,843
COJO	Bovine leukemia virus susceptibility	1.74E-02	2	2	23:22,556,761; 23:27,294,315

GxE - day length	Metabolic body weight	6.12E-05	115	8	3:9,874,711; 14:1,073,049; 16:52,306,052; 20:4,609,314; 20:4,617,648; 20:4,633,738; 20:4,653,700; 20:4,655,604
GxE - day length	Ketosis	4.18E-03	21	13	3:32,993,574; 9:64,290,905; 13:52,556,307; 14:796,638; 14:1,073,049; 20:58,688,893; 20:58,742,109; 20:58,745,645; 20:58,748,682; 20:58,762,246; 20:58,792,088; 20:58,796,066; 20:59,139,410
GxE - day length	Lifetime profit index	8.47E-03	6	2	14:796,638; 14:1,073,049
